# Enhancing Fatigue Lifetime of Secondary AlZn10Si8Mg Alloys Through Shot Peening: Influence of Iron Content and Surface Defects

**DOI:** 10.3390/ma18163901

**Published:** 2025-08-20

**Authors:** Denisa Straková, Zuzana Šurdová, Eva Tillová, Lenka Kuchariková, Martin Mikolajčík, Denisa Závodská, Mario Guagliano

**Affiliations:** 1 Department of Materials Engineering, Faculty of Mechanical Engineering, University of Zilina, Univerzitna 8215/1, 010 26 Zilina, Slovakia; zuzana.surdova@fstroj.uniza.sk (Z.Š.); eva.tillova@fstroj.uniza.sk (E.T.); lenka.kucharikova@fstroj.uniza.sk (L.K.); martin.mikolajcik@fstroj.uniza.sk (M.M.); 2 VUTCH, Rybniky 954/10, 011 68 Zilina, Slovakia; zavodska@vutch.sk; 3 Department of Mechanical Engineering, Politecnico di Milano, 20158 Milano, Italy; mario.guagliano@polimi.it

**Keywords:** secondary aluminium alloys, shot peening, fatigue properties, iron content, AlZn10Si8Mg

## Abstract

The rising demand for aluminium and environmental concerns highlight the need for a circular economy using recycled alloys. This study examines the effect of shot peening on the high-cycle fatigue life of secondary AlZn10Si8Mg alloys with different iron contents: Alloy A (0.14 wt.% Fe) and Alloy B (0.56 wt.% Fe). Although both alloys showed similar tensile properties, Alloy B had higher porosity and finer β-Al_5_FeSi intermetallics. Shot peening was applied at 100% and 1000% coverage to evaluate changes in surface roughness, porosity, residual stresses, and fatigue performance. The treatment significantly reduced surface-connected porosity via plastic deformation, enhancing fatigue life despite increased roughness. Fatigue tests showed a 21% increase in fatigue limit for Alloy A and a 6% gain for Alloy B at higher coverage. Fractographic analysis revealed that 95% of fatigue cracks initiated at surface pores. Residual stress measurements confirmed compressive stresses were limited to the near-surface layer, with minimal influence on subsurface crack propagation. Overall, shot peening proves to be an effective method for improving fatigue resistance in recycled aluminium alloys, even in alloys with elevated iron content, reinforcing their potential for structural applications under cyclic loading.

## 1. Introduction

The increasing global demand for aluminium, driven by its widespread use in automotive, aerospace, and construction industries, has heightened the need for sustainable production practices. Transitioning to a circular economy, where secondary (recycled) aluminium alloys replace primary production, offers significant environmental and economic benefits, including energy savings and reduced greenhouse gas emissions [1,2,3,4,5].

However, aluminium recycling presents metallurgical challenges due to the presence of impurities such as iron, which are difficult to eliminate. Elevated iron content promotes the formation of hard and brittle intermetallic phases, notably β-Al_5_FeSi, which degrade mechanical performance by acting as stress concentrators and fatigue crack initiation sites [6,7,8,9,10,11,12]. These phases are often associated with increased porosity during solidification, further impairing the fatigue properties of cast components [12,13,14,15].

To address this, the use of self-hardening Al-Zn-Si-Mg alloys has gained attention. These alloys develop desirable mechanical properties through natural ageing at room temperature, eliminating the need for conventional T6 heat treatment [16,17,18,19]. This makes them particularly attractive for high-throughput, energy-efficient manufacturing. While self-hardening behaviour results in fine, densely distributed precipitates that enhance strength [20,21,22], their fatigue resistance remains sensitive to microstructural heterogeneities such as Fe-rich intermetallics and casting defects.

Surface treatment methods, such as shot peening, offer a promising solution for improving fatigue life by inducing compressive residual stresses, closing surface-connected porosity, and increasing resistance to crack initiation [23,24,25,26,27]. This method has demonstrated efficacy in various aluminium alloys by enhancing near-surface properties and delaying fatigue crack nucleation, especially in components with surface-adjacent defects.

Despite these advantages, the combined influence of iron content, intermetallic morphology, and shot peening coverage levels on fatigue resistance in secondary AlZn10Si8Mg alloys is not well established. A deeper understanding of these relationships is essential for advancing the use of recycled aluminium alloys in structural components subject to cyclic loading.

This study investigates the effects of shot peening on the fatigue life of secondary AlZn10Si8Mg alloys with two distinct iron contents (0.14 wt.% and 0.56 wt.%). Surface porosity, microstructure, and residual stress profiles are examined for their contribution to fatigue behaviour. By analysing the microstructure, mechanical properties, and fracture mechanisms, this work provides insights into how shot peening can enable broader application of secondary aluminium alloys in demanding engineering environments.

## 2. Experimental Procedures

### 2.1. Material and Specimen Preparation

Cylindrical test bars with a diameter of 20 mm and a length of 300 mm were produced from self-hardening secondary aluminium alloy AlZn10Si8Mg using a sand-casting process without any modification or refinement. Two variants with different iron contents were created: secondary Alloy A with 0.14 wt.% Fe and secondary Alloy B with 0.56 wt.% Fe. Stringent process control measures were employed to minimise contamination risk during melting, including carefully monitoring temperature, flux addition, and alloy addition. The chemical composition of the experimental alloys was determined using Optical Emission Spectroscopy (OES), and the results are presented in Table 1. The EN-AC-71100 standard [28] specifies a maximum iron content in these alloys of up to 0.3%. However, since iron is tough to remove during recycling [9], this paper aims to verify whether alloys with a higher iron content can be used.

### 2.2. Shot Peening and Surface Roughness Measurement

**Figure 1:**
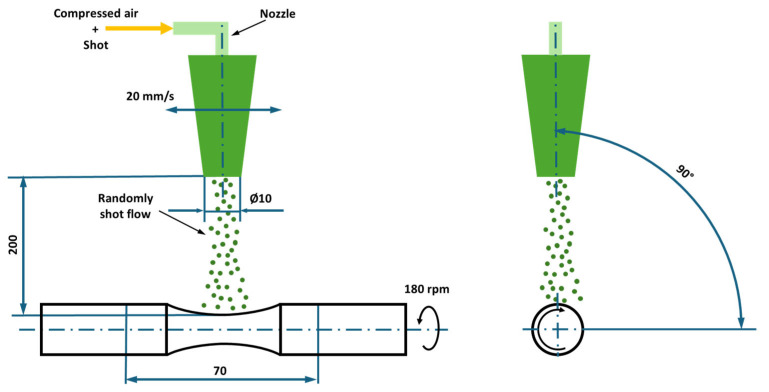
Schematic illustration of the shot peening process performed on hourglass fatigue test bars [29].

To determine the impact of shot peening on the fatigue limit, 15 test bars of Alloy A and 15 of Alloy B were subjected to shot peening with 100% coverage, termed SP100. For further comparison, an additional 15 test bars of Alloy A and 15 of Alloy B were shot peened with 1000% coverage, termed SP1000 (Figure 1). Coverage refers to the percentage of the surface area impacted during the shot peening process. Coverage beyond 100% is achieved by prolonging the peening duration. To ensure reproducibility and comparability of the measurements, test bars were shot peened according to parameters specified in Table 2.

Surface roughness was evaluated using an Alicona G5 Infinite Focus optical 3D measurement microscope (Alicona Imaging GmbH, Graz, Austria) following the ISO 4287 standard [30]. Average values of standard roughness parameters, such as the arithmetic mean of the absolute ordinates (R_a_), as well as the average values of the absolute heights of the five highest-profile peaks and the depths of the five deepest valleys within the evaluation length (R_z_) from the reference line of the roughness profile, were reported for each experimental alloy.

### 2.3. Tensile Strength Test

**Figure 2:**
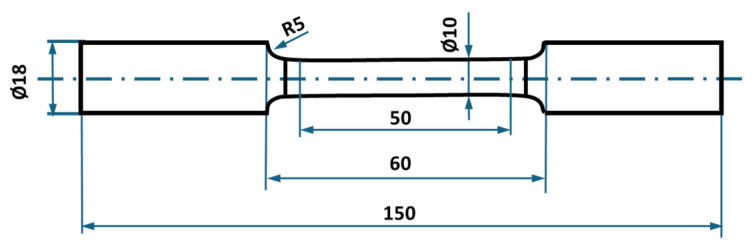
Tensile test bar dimensions according to the ISO 6892-1 standard [31] (dimensions given in mm).

Tensile testing was conducted on secondary aluminium Alloys A and B, represented by three specimens. Test bars were machined into specimen dimensions (Figure 2) according to the ISO 6892-1 standard [31]. Test bars were tested on an MTS Alliance RT/100 device at a strain rate of 0.7 mm/min, up to 2% strain, followed by a constant rate of 2 mm/min.

### 2.4. Microstructural Characterisation

Specimens for microstructural analysis were prepared from test bars according to ASTM E3 metallographic testing standards [32] used for aluminium alloys. The microstructure was revealed on polished cross-sections etched with 0.5% HF (CentralChem, Bratislava, Slovakia). The microstructure was analysed using an optical microscope, NEOPHOT 32 (Carl Zeiss Jena GmbH, Jena, Germany), with NIS Elements software, version 4.20 (Nikon Corporation, Tokyo, Japan), covering quantitative measurements of casting defects. Thermo-Calc software (Thermo-Calc Software AB, Stockholm, Sweden) and the COST2 database (Thermo-Calc Software AB, Stockholm, Sweden) were used to conduct thermodynamic calculations to investigate the phase evolution. The phase equilibria of the system were calculated within the temperature range of 0–600 °C. The calculation considered elements P, N, Cu, Mg, Mn, Zn, Si, and Fe and the presence of different phases, including liquid, β-AlFeSi (monoclinic), eutectic Si (cubic diamond-A4), Laves phase MgZn_2_ (hexagonal), α-Al (matrix FCC-A1), Mg_2_Si (cubic), α-AlMnSi (cubic), Mg_2_Zn_11_ (cubic), Zn (hexagonal), θ-Al_2_Cu (tetragonal), Al_6_Mn (orthorhombic), and α-AlFeSi (cubic). Porosity was examined with a CT scanner ZEISS METROTOM (Carl Zeiss Industrielle Messtechnik GmbH, Oberkochen, Germany), and microfractographic examination was done using SEM TESCAN VEGA LMU II (TESCAN ORSAY HOLDING, a.s., Brno, Czech Republic).

### 2.5. Hardness and Microhardness Measurements

Brinell hardness testing followed the ISO 6506-1 standard [33] using a NEXUS 3000 device (Innovatest Europe BV, Maastricht, The Netherlands). Hardness measurements were performed on polished cross-sections of the experimental samples, applying a load of 250 kgf with a dwell time of 15 s for each indentation. Five measurements were taken for each sample (Alloys A and B). Additionally, microhardness measurements were carried out following the EN ISO 6507-1 standard [34], using a ZwickRoell hardness tester (ZwickRoell GmbH & Co. KG, Ulm, Germany). Ten measurements were taken for the eutectic and *α*-matrix (HV 0.025) in Alloys A and B.

### 2.6. Fatigue Tests and Fractography Analysis

**Figure 3:**
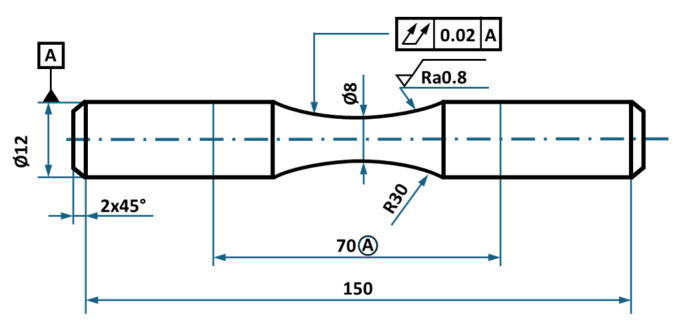
Fatigue test bar with hourglass geometry according to ISO 12,107 standard [35] (dimensions given in mm).

The effect of iron content on fatigue lifetime was studied by subjecting Alloys A and B to shot peening at different coverage levels (100% and 1000%), building on [15] investigation of un-peened alloys. Fatigue testing followed the ISO 12107 standard [35], employing rotation-bending at a 30 Hz frequency and load ratio R = −1. Each alloy group, comprising 15 specimens, was tested at room temperature. Test bars were machined into an hourglass shape (Figure 3) with a fillet radius sufficient to prevent notch-related fatigue phenomena. Fractographic analysis was performed on the fractured surfaces of the test bars using a Zeiss EVO50 scanning electron microscope (Carl Zeiss AG, Oberkochen, Germany).

### 2.7. Residual Stress Measurement

To evaluate the contribution of surface residual stresses to fatigue life, X-ray diffraction (XRD) was used to measure the stress distribution in shot peened specimens. The underlying hypothesis is that compressive residual stresses induced by shot peening delay fatigue crack initiation by suppressing tensile stress concentrations at surface-connected defects, thereby extending fatigue life. Conversely, if residual stress relaxation or shallow compressive zones occur, their contribution to fatigue resistance may be limited.

Residual stresses were assessed using an AST X-Stress 3000 portable X-ray diffractometer (Stresstech Oy, Jyväskylä, Finland) with CrKα radiation (λKα_1_ = 2.2898 Å), applying the sin^2^(ψ) method and targeting the 311 diffraction peak at a 2θ angle of 139°. The measurement protocol involved scanning seven tilt angles from −45° to +45° across three rotation directions (0°, 45°, and 90°) with a constant step size of 0.028°. Prior to data acquisition, system calibration was validated using a standard polycrystalline Al reference sample.

To obtain depth profiles of residual stress, successive surface layers (2 × 2 mm) were incrementally removed by electro-polishing. A solution of 94% CH_3_COOH and 6% HClO_4_ (CentralChem, Bratislava, Slovakia) was used at 35 V, enabling non-destructive layer removal with minimal alteration of the underlying stress state. The evolution of stress with depth was used to estimate the effective penetration of compressive stress and to relate these findings to crack initiation zones observed in fractographic analysis.

## 3. Results

### 3.1. Tensile Testing and Hardness

Tensile tests revealed similar mechanical properties between the two secondary AlZn10Si8Mg alloys despite their differing iron contents (Table 3). Alloy A (0.14 wt.% Fe) and Alloy B (0.56 wt.% Fe) both exhibited ultimate tensile strengths around 180 MPa and elongations to failure of 2%. Hardness values (HB and HV) also remained largely unchanged between the alloys, although a notable microhardness contrast was observed between the α-Al matrix and eutectic Si. The latter phase demonstrated ~50 HV higher hardness due to its covalent bonding and diamond cubic structure. These findings are consistent with prior studies showing that local hardness variations in eutectic networks can act as microstructural barriers to dislocation movement [18,22]. However, their role in fatigue is not only governed by hardness but also by spatial distribution and morphology.

### 3.2. Microstructure Analysis

**Figure 4:**
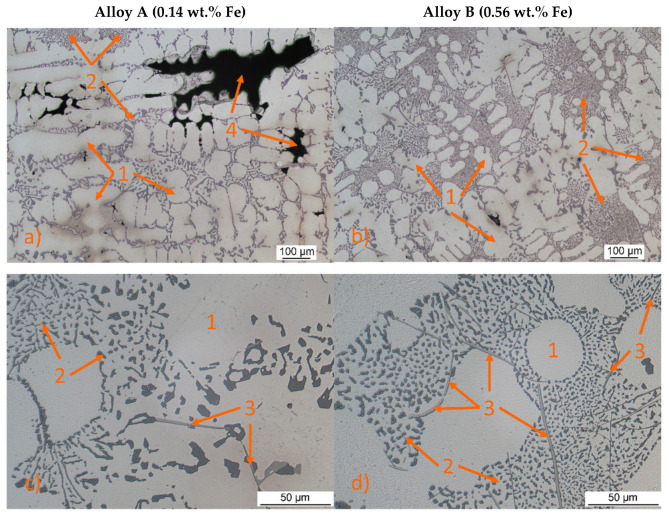
OM microstructures of Alloy A and Alloy B with different iron content, etch. 0.5% HF: (**a**) Alloy A; (**b**) Alloy B; (**c**) detail of Alloy A; (**d**) detail of Alloy B (1—α-phase; 2—eutectic; 3—Al_5_FeSi; 4—shrinkage porosity).

Both secondary aluminium alloys A and B, as shown in Figure 4, exhibited similar microstructures consisting of α-phase (Figure 4—no. 1), eutectic Si crystals (Figure 4—no. 2), and various intermetallic phases, including Mg_2_Si, β-Al_5_FeSi needles (Figure 4—no. 3), and ternary eutectic Al-MgZn_2_-Cu) phases. The Al-MgZn_2_-Cu and Mg_2_Si phases were only visible in minor proportions and did not significantly impact the overall properties. The α-phase, a substitutional solid solution of Zn in Al, appeared in the form of dendrites. Eutectic silicon particles were observed as small, poorly rounded grains located mainly at the periphery of α-phase dendrites. Eutectic silicon crystals appear to be finer in the centre of the eutectic. At the eutectic/matrix interface they are more angular and larger, which can lead to stress localization.

The Fe phases manifested as β-Al_5_FeSi needles, with Alloy A (0.14 wt.% Fe) showing slightly coarser eutectic silicon and shorter, thinner β-Al_5_FeSi phases compared to Alloy B (0.56 wt.% Fe). Previous work has shown that β-Al_5_FeSi, due to its brittle and elongated morphology, significantly contributes to fatigue crack initiation [10,11,36,37]. These phases are often located near eutectic regions, and when aligned with eutectic silicon and pores, can form connected networks that facilitate early-stage crack propagation. Thus, the combined effect of pore morphology, Fe-rich intermetallics, and eutectic Si distribution needs to be considered for an accurate fatigue assessment. The microstructure also revealed shrinkage porosity (Figure 4—no. 4) in regions where solidification was last to occur, such as thick sections, junctions, and regions farthest from the cooling source. These areas are prone to shrinkage defects due to insufficient feeding of molten metal during solidification, resulting in voids or pores [38].

**Figure 5:**
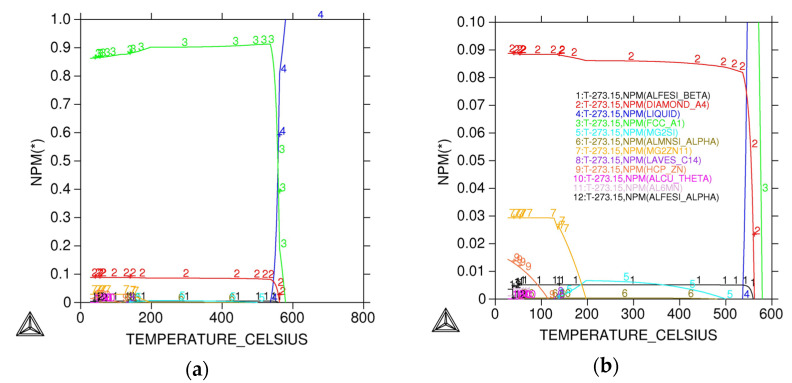
(**a**) Equilibrium phase constituents predicted by Thermo-Calc software for the designed Alloy A chemistry; (**b**) detail of (**a**).

Equilibrium phase diagrams (Figure 5) were constructed to illustrate the phase evolution with temperature for Alloy A, revealing typical phases including *α*-Al (matrix), eutectic Si, Al_5_FeSi, Mg_2_Si, and AlMgZn_2_Cu. Aluminium nucleation was observed at 580 °C, followed by silicon nucleation at 570 °C. Solidification and phase growth simulations elucidated a sequential formation of phases: (Al) + *β*-Al_5_FeSi → (Al) + *β*-Al_5_FeSi + Mg_2_Si → (Al) + *β*-Al_5_FeSi + Mg_2_Si + AlMgZn_2_Cu. The β-Al_5_FeSi phase solidified first at 550 °C followed by Mg_2_Si at 500 °C and AlMgZn_2_Cu at 200 °C.

**Figure 6:**
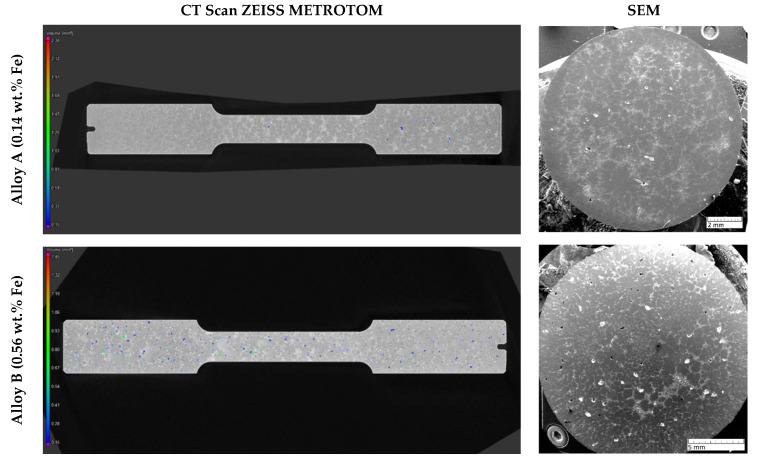
Quantification of pores using CT scan and SEM analysis, etch. 0.5% HF.

**Figure 7:**
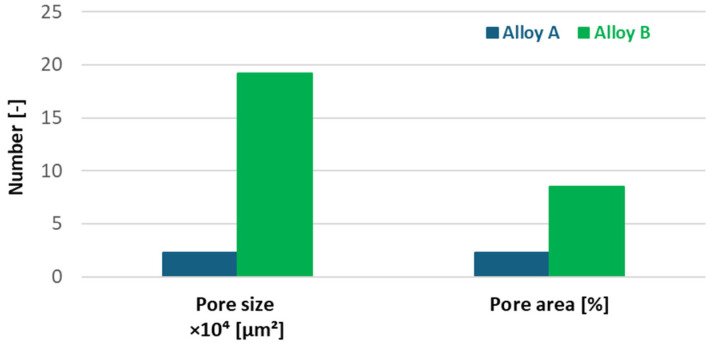
Quantitative analysis of secondary aluminium Alloys A and B.

Increasing iron content in the experimental alloys significantly enhances porosity. Alloy B exhibited larger, more widely distributed pores than Alloy A, increasing total pore area from 2.26% to 8.5% (Figure 6; Figure 7). Porosity, particularly when irregular in shape or located near hard phases, amplifies stress concentrations. As shown in prior work [38], such shrinkage porosity acts as a dominant fatigue crack nucleation site in cast aluminium alloys.

**Figure 8:**
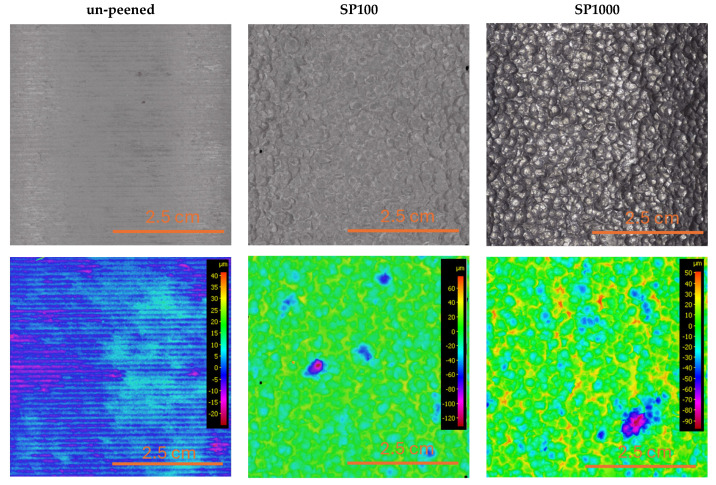
Surface topology after shot peening with different coverage levels.

**Figure 9:**
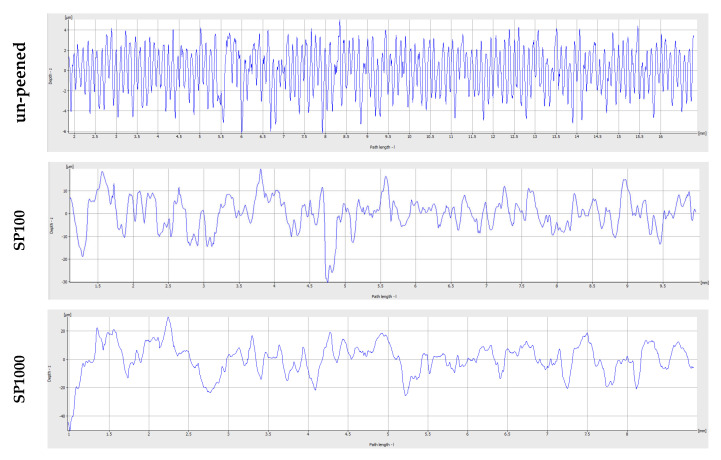
Surface roughness profiles of un-peened samples and samples after shot peening, varying by coverage levels (λ_c_ = 2500 μm).

Figure 8 shows the top surface microstructures of Alloy A in the un-peened (machined) condition and after shot peening at 100% and 1000% coverage. Despite identical chemical composition across all samples, notable differences in surface morphology emerged due to mechanical treatment. Shot peening introduced substantial plastic deformation, visibly altering the surface and partially closing existing pores. This localized densification likely contributed to reduced stress concentrations at defect sites. Corresponding surface roughness profiles (Figure 9) confirmed a progressive increase in roughness with higher peening coverage. The un-peened sample exhibited an R_a_ of 1.8 μm (R_z_ = 9.9 μm), which increased to 5.78 μm (R_z_ = 33.7 μm) at 100% and to 8.57 μm (R_z_ = 47.6 μm) at 1000% coverage. Although increased surface roughness is typically associated with earlier crack initiation, the observed improvement in fatigue life suggests that the beneficial effects of pore compression and induced compressive residual stress outweigh the potential drawbacks of surface topography.

### 3.3. High-Cycle Fatigue Strength and Residual Stress Analysis

**Figure 10:**
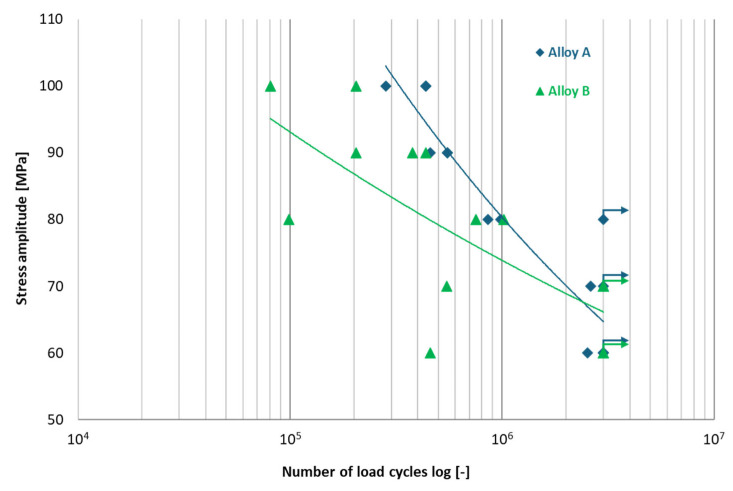
Effect of different Fe content on fatigue lifetime in secondary aluminium Alloys A and B for load/stress ratio *R* = −1 [15].

The high-cycle fatigue strength results for Alloys A and B were previously published in [15] research, with the test results illustrated in Figure 10. An S-N curve was constructed, indicating the fatigue limit for Alloy A at $\sigma_{{c3\times 10}^{6}}$ = 65 MPa and for Alloy B at $\sigma_{{c3\times 10}^{6}}$ = 66 MPa. Markers with arrows indicate specimens that did not fail before reaching the cycle limit (run-out). Despite a higher percentage of Fe, the results indicated that specimens exhibited extended fatigue life, despite elevated iron content. Building on this research, samples of Alloys A and B were shot peened at different coverage levels and underwent high-cycle fatigue testing under the same conditions as in the [15] study.

**Figure 11:**
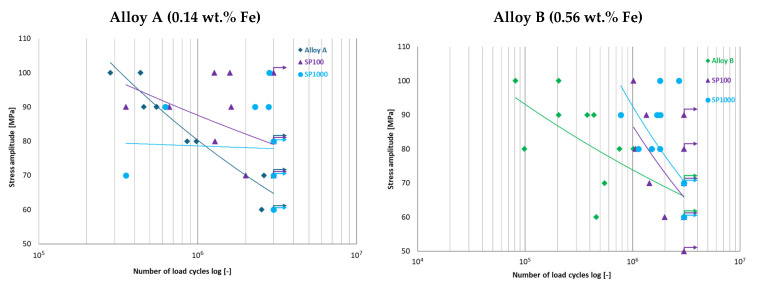
Comparison of S-N curves following shot peening with 100% and 1000% coverage of secondary aluminium Alloys A and B.

Fatigue testing confirmed the detrimental effect of increased Fe content on fatigue resistance. However, shot peening improved the fatigue limit of both alloys. Alloy A showed a fatigue limit increase from $\sigma_{{c3\times 10}^{6}}$ = 65 MPa (un-peened) to 79 MPa (SP100) and 78 MPa (SP1000), while Alloy B improved from 66 MPa to 70 MPa under SP1000 treatment (Figure 11). The more modest improvement in Alloy B is likely due to its higher initial defect density and the presence of more pronounced intermetallics. To provide a clearer comparison of the fatigue performance, Table 4 summarizes the experimentally determined fatigue limits of the two studied alloys under different surface conditions.

**Figure 12:**
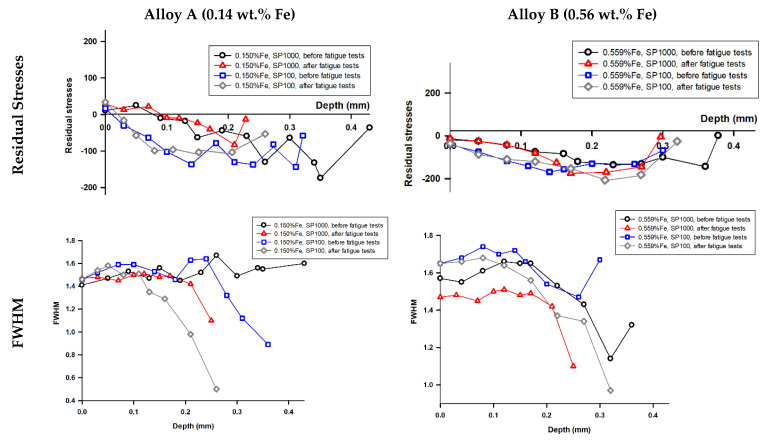
Distribution of residual stresses and FWHM before and after fatigue tests measured at φ = 90°.

Given the interplay between residual stress and fatigue behaviour, the distribution of residual stress in the experimental alloys was measured. Figure 12 presents residual stress assessment results in secondary aluminium Alloys A and B after shot peening for both SP100 and SP1000 treatments. The beneficial effects of shot peening are primarily attributed to compressive residual stresses and pore closure. Compressive surface stresses are known to suppress microcrack nucleation, and this effect was confirmed here. However, their effectiveness is limited by penetration depth (~0.4 mm), as also shown in residual stress profiles (Figure 12). These residual stresses do not significantly influence fatigue crack initiation in regions beyond this depth, where pores and Fe phases are more crucial. Increasing the peening coverage level from 100% to 1000% did not enhance the residual stress field; the stored energy, represented by the area under the Full Width at Half Maximum (FWHM) versus depth curves, remained nearly identical. The FWHM decreased with depth, indicating diminished work hardening and residual stress effects beyond 0.4 mm below the original surface. This suggests that the shot peening process had no significant impact beyond this depth.

**Figure 13:**
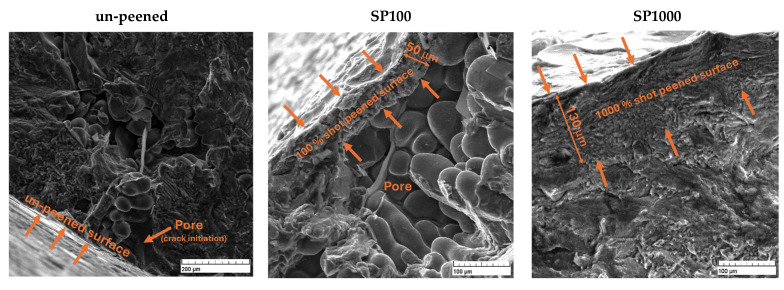
Fractographic analysis of the area near the surface after fatigue testing comparing un-peened and shot peened samples with different coverage levels.

Fatigue cracks in un-peened Alloys A and B samples were primarily initiated from casting defects, such as pores or shrinkage cavities, as illustrated in Figure 13. These defects are typically located near or at the specimen’s free surface. The shot peening process effectively mitigated these issues by visibly reducing surface porosity, minimizing pore size, and decreasing their overall number. Surface pores were visually reduced by shot peening, as seen in Figure 13, supporting the conclusion that mechanical densification helps to eliminate critical crack initiation sites. While surface roughness increased post-peening, its negative impact was outweighed by the benefits of residual stress and porosity reduction, in agreement with observations from [27] on fretting fatigue. This improvement positively impacted the fatigue lifetime of the materials. The thickness of the shot peened layer varied with different levels of shot peening coverage. For SP100, the layer thickness averaged approximately 50 μm, whereas, for SP1000, it increased to about 130 μm. Despite these variations, fatigue cracks still initiated from the surface, albeit at significantly fewer locations due to the treatment.

In summary, the fatigue lifetime of recycled AlZn10Si8Mg alloys is strongly governed by the interplay of porosity, Fe-rich intermetallics, and eutectic Si morphology. Shot peening is shown to be a highly effective method to mitigate these effects, but its success is limited when defect size and distribution exceed the depth of stress penetration. These findings underscore the importance of integrating microstructural control during casting with post-process surface treatments to optimize fatigue performance in recycled aluminium components.

## 4. Discussion

The present study demonstrates that shot peening significantly enhances the fatigue resistance of recycled AlZn10Si8Mg alloys, especially in conditions where surface-connected defects are dominant. The primary mechanism responsible for the improvement is the local plastic deformation and densification induced by shot peening, which closes surface pores and introduces beneficial compressive residual stresses.

Alloy A, with lower Fe content, exhibited a more pronounced improvement in fatigue performance due to a lower initial porosity level and reduced presence of brittle intermetallics. The data clearly show that the beneficial effects of shot peening saturate at 100% coverage, with minimal gains observed at 1000% coverage. This indicates that 100% coverage represents an optimal trade-off between fatigue performance and processing cost.

The results confirm that surface porosity, more than bulk microstructure, governs crack initiation in both alloys. Fractography supports this, showing 95% of failures initiating at surface pores. While increased surface roughness is traditionally seen as detrimental to fatigue, in this case its negative influence was outweighed by the suppression of critical defect-driven stress concentrations.

The implications of these findings are twofold. First, they provide strong evidence that recycled aluminium alloys, even those exceeding standard Fe thresholds, can meet the fatigue requirements of structural applications when post-processed appropriately. Second, the results inform process optimization: shot peening must be carefully tuned to balance mechanical enhancement against roughness-related risks.

Overall, the study highlights that surface engineering, more than alloy chemistry, can enable the use of recycled AlZn10Si8Mg alloys in fatigue-critical applications such as automotive suspension or structural fasteners.

## 5. Conclusions

This study demonstrates that shot peening significantly enhances the high-cycle fatigue life of secondary AlZn10Si8Mg alloys, even in the presence of elevated iron content and associated casting defects. The key findings and their implications are as follows:

- Increasing the iron content from 0.14 wt.% (Alloy A) to 0.56 wt.% (Alloy B) led to a substantial rise in total porosity (from 2.26% to 8.5%) as well as a shift toward more irregular pore morphologies. While tensile and hardness properties remained similar for both alloys, Alloy B showed a higher prevalence of large, non-spherical pores with sharp edges, which serve as potent stress concentrators and primary sites for fatigue crack initiation. In addition to the increased porosity, the elevated Fe content promoted the formation of needle-like β-Al_5_FeSi intermetallics. These brittle, elongated phases are unevenly distributed and tend to form colonies near eutectic regions, further intensifying local stress concentrations. Their sharp morphology and preferential orientation may not only facilitate crack initiation but also promote crack propagation under cyclic loading. Moreover, the spatial distribution of eutectic Si crystals—often clustered around α-phase dendrite boundaries—can influence fatigue behaviour by introducing additional heterogeneities in the microstructure. Although eutectic Si exhibits significantly higher hardness (~50 HV more than α-Al), its irregular and often angular morphology, particularly at eutectic/matrix interfaces, may contribute to stress localization and facilitate early-stage microcrack formation. This effect becomes more pronounced when coupled with adjacent Fe-rich intermetallics and casting pores. Together, the presence of irregularly shaped pores, β-Al_5_FeSi phases, and unfavourably distributed eutectic Si contributes to enhanced local strain accumulation, reduced fatigue resistance, and earlier failure in Alloy B despite its comparable static mechanical properties.
- Shot peening effectively mitigated the detrimental influence of surface-connected porosity, primarily through plastic deformation and partial pore closure. This resulted in a measurable increase in fatigue limit—up to 21% in Alloy A and 6% in Alloy B—despite the associated increase in surface roughness. These improvements are in line with, or exceed, fatigue strength increases reported for wrought and cast aluminium alloys subjected to shot peening, where typical gains range from 10% to 20% under comparable conditions [15,27,28]. This suggests that secondary alloys, despite their elevated impurity levels, can benefit significantly from surface engineering treatments.
- Residual stress analysis confirmed that compressive stresses induced by shot peening were confined to near-surface regions (~0.4 mm). While these stresses did not significantly alter subsurface crack propagation, they contributed to delayed crack initiation by counteracting surface tensile loading.
- Fractographic evidence revealed that 95% of fatigue cracks originated at surface pores, highlighting the importance of surface condition in fatigue life. The effectiveness of shot peening in improving fatigue resistance was closely linked to the ability to reduce these critical surface defects.

The results confirm that secondary aluminium alloys with elevated iron content can achieve fatigue life suitable for structural applications when appropriately post-processed. Shot peening emerges as a cost-effective and scalable surface treatment that enables broader deployment of recycled alloys in weight-sensitive, fatigue-critical components such as automotive castings and structural fasteners.

## Figures and Tables

**Table 1:** Chemical composition [wt.%] of AlZn10Si8Mg with different Fe content.

	Fe	Si	Mn	Ti	Cu	Mg	Zn	Ni	Bi	Sb	Al
**Alloy A**	0.14	8.7	0.13	0.07	0.003	0.38	8.7	0.04	0.003	0.008	Bal.
**Alloy B**	0.56	9.1	0.19	0.07	0.010	0.31	8.1	0.002	0.004	0.008	Bal.

**Table 2:** Shot peening parameters.

Peening Media (PM)	PM Material	PM Diameter	PM Hardness	Almen Intensity	Angle of Impingement	Coverage
S170H	steel	425 μm	45 HRC	12A	90°	100% 1000%

**Table 3:** Mechanical properties of experimental secondary aluminium alloys.

		UTS [MPa]	A [%]	HB	HV 0.025 (α-Matrix)	HV 0.025 (Eutectic Si)
**Alloy A**	**un-peened**	187	2	83	82	134
**Alloy B**	**un-peened**	178	2	86	82	136

**Table 4:** Fatigue limit $\;\sigma_{{c3\times 10}^{6}}$ of AlZn10Si8Mg alloys with different Fe content and shot peening coverage levels.

Alloy	Fe Intent [wt.%]	Surface Condition	$\mathrm{Fatigue}\;\mathrm{Limit}\;\sigma_{{c3\times 10}^{6}}$[MPa]
Alloy A	0.14	un-peened	65
Alloy A	0.14	SP100	79
Alloy A	0.14	SP1000	78
Alloy B	0.56	un-peened	66
Alloy B	0.56	SP100	66
Alloy B	0.56	SP1000	70

## Data Availability

The original contributions presented in this study are included in the article. Further inquiries can be directed to the corresponding authors.
